# Matrix metalloproteinases and genetic mouse models in cancer research: a mini-review

**DOI:** 10.1007/s13277-014-2747-6

**Published:** 2014-10-29

**Authors:** Edyta Wieczorek, Ewa Jablonska, Wojciech Wasowicz, Edyta Reszka

**Affiliations:** Department of Toxicology and Carcinogenesis, Nofer Institute of Occupational Medicine, Lodz, Poland

**Keywords:** MMP, Cancer, Genetic mouse models, Genetic polymorphism, Association studies, Susceptibility

## Abstract

Carcinogenesis is a multistep and also a multifactorial process that involves agents like genetic and environmental factors. Matrix metalloproteinases (MMPs) are major proteolytic enzymes which are involved in cancer cell migration, invasion, and metastasis. Genetic variations in genes encoding the MMPs were shown in human studies to influence cancer risk and phenotypic features of a tumor. The complex role of MMPs seems to be important in the mechanism of carcinogenesis, but it is not well recognized. Rodent studies concentrated particularly on the better understanding of the biological functions of the MMPs and their impact on the pathological process, also through the modification of *Mmp* genes. This review presents current knowledge and the existing evidence on the importance of selected MMPs in genetic mouse models of cancer and human genetic association studies. Further, this work can be useful for scientists studying the role of the genetic impact of MMPs in carcinogenesis.

## Introduction

The interactions between tumor cells and their microenvironment reveal the key role of matrix metalloproteinases (MMPs) during the process of carcinogenesis. Tumor growth and metastasis formation depend on the cell-cell and cell-matrix interactions and also modifications of the tissue through the action of proteolytic enzymes [1–3]. In the early 1980s, Liotta et al. indicated in in vitro studies on mouse cancer cells the importance of these enzymes in the process of metastasis [4]. Members of the MMP family of extracellular proteases include six subgroups which, due to differences in their structure domain, may be involved in a large variety of physiological and pathological processes [5]. The large family of MMPs is composed of 25 endopeptidases in humans and 24 in mice [6, 7]. MMP enzymes play a significant role in cancer invasion, metastasis, and angiogenesis also through their impact on cell behavior such as growth of metastasized tumor cells and increased motility of the epithelial cells [5, 8]. Studies show that the activity of MMP plays a role in extracellular matrix (ECM) protein breakdown, cleavage of cell surface receptors, and release of apoptotic signals, and it is associated with advanced stages and poor clinical outcome in various types of cancer [9–11]. However, MMPs have been reported to be important also at an early stage of tumor progression [12–14]. In 2003, Balbin et al. in his study demonstrated for the first time that *Mmp-8* has a protective role in mouse skin cancer [15]. Nowadays, the reviews focused on the MMP role in cancer metastasis and angiogenesis postulated various opposed effects, such as tumor supporting and inhibiting [16–18]. In this paper, we shall present and discuss current knowledge about MMP studied in mice tumor models and human genetic association studies.

## Evaluation of the role of MMP in cancer in genetic mouse models

The design and conduction of genetic association studies can be challenging and replete with difficulties because of the balance of different risks in relation to one another. For all these reasons, the mouse model studies allow us to control or eliminate the effects of genetic and environmental variation and enhance our understanding of cancer disease [19–21]. Despite several limitations resulting from the ethical and technical constraints, the mouse model is a valuable tool because some types of tumors in mice are similar in morphology, histopathology, and molecular characteristics to human tumors [22]. Nowadays, there are many ways to generate genetically engineered mice (GEM)—mice with induced mutations, such as mouse loss of function, i.e., knockdown, knockout, and dominant negative, and mouse gain of function, i.e., transgenic, knockin, and virus-mediated delivery [19, 23]. The *Mmp* gene knockout mutation also known as *Mmp-*deficient or null mice is the most common among GEM. Mouse mutants increased our understanding of the molecular and biological functions of protein by engineering constitutive or conditional deletions which delete or invert all or part of a target gene such that the gene is inactivated [24]. Because the loss of function of a gene may be comparable to the effect that occurs for the functional genetic polymorphism in humans, they can be used as a background for showing a relationship between genotype and cancer risk factor.

Genetic mouse models reveal the complexity of MMPs in a variety of biological processes together with normal and pathological tissue conditions, under identical environmental and genetic conditions [25, 26]. Therefore, they are useful in extending our understanding of cancer pathogenesis. The use of combined mutational mouse models may be exploited to demonstrate interactions between MMPs and other ECM molecules important in understanding the carcinogenic mechanism [17, 27]. Interactions between MMPs and proteinases of other classes are another important aspect of tumor biology. Understanding these interactions is also necessary for development of effective therapeutic strategies [28]. Additionally, in vitro studies in mice bring us closer to understanding not only the role of MMPs but also the importance of genetic variants of *Mmp* genes. Thus, genetically modified mouse models allow us to explore the mechanisms underlying the role of various MMPs in cancer, which may be helpful in planning and interpreting future human genetic association studies.

The MMP mRNA expression and activity determined by real-time PCR, zymography, immunoblotting, or immunohistochemistry are recently used as biomarkers of tumor invasion and metastasis in mice, as shown by numerous in vivo studies [29–32].

## Genetic polymorphisms in the *MMPs* as a regulator of *MMP* gene expression and their impact on cancer risk

The synthesis of the MMPs is observed under both normal and pathological conditions [33]. Connective tissue cells such as fibroblasts, leukocytes, and macrophages, and tumor stromal elements can synthesize and secrete proMMPs. A wide variety of extracellular factors, including specific tissue MMP inhibitors, cytokines, and environmental growth factors, regulate the synthesis and activity of MMPs in tissue. Expression of *MMP* genes is under transcriptional regulation by the extracellular factors, transcription factors such as activator protein-1, E-twenty-six specific domain, Sp-1, nuclear factor-κB, and promoter TATA box, and also under regulation of genetic polymorphisms [34–36].

The genetic polymorphisms are DNA heritable sequence variants in the genome that may contribute to phenotypic variability, which cause variation in expression, including silencing of genes. Most of the common genetic variants are single nucleotide polymorphisms (SNPs) and deletion-insertion variants (DIVs). These changes probably represent the majority of genetic variability in the human population [37]. A SNP consists in base pair substitution, while a DIV involves nucleotide deletion or insertion. Those allelic variants generated as the result of conversion of a nucleotide to another at a homologous position in the promoter region of the gene may affect gene transcriptional activity. SNPs in the promoter region of *MMP* may affect transcription through creating a binding site for E-twenty-six or abolishing the binding site for Sp1 [38, 39]. At the same time, SNPs located in exons may lead to a replacement and affect protein function. Another type of genetic polymorphisms is polymorphic microsatellites containing cytosine-adenine dinucleotide repeats and rare copy number variations (CNVs). Taken together, these genetic variabilities have a proven or likely effect on gene expression with a possible impact on the process of tumorigenesis and cancer risk [40].

The functional polymorphisms in the *MMPs* have been examined in many cancer-associated studies among various populations. Those studies investigated not only the relationship between common genetic polymorphisms and cancer risk but also cancer prognosis, invasiveness, and recurrence [41–43]. The resultant data confirm not only the impact of various genetic polymorphisms in the *MMPs* on cancer risk but also the lack of association. The results appear to be unclear about whether some common genetic polymorphisms of *MMPs* may be used as a predictor of cancer risk. Thus, the goals of meta-analysis were to provide an overview of the evidence regarding the *MMP* genetic polymorphisms and cancer risk [44–48].

To date, genome-wide association studies which used only total cases of cancer versus the control population have not identified loci in MMPs to affect breast or bladder cancer risk [49, 50]. Also, studies which use mouse mapping quantitative trait loci to predicted human disease *MMP* genes have not been identified [51, 52]. Both methods have limitations, but use of the GEM might enable better understanding of these results.

Most reports agree that factors contributing to cancer development involve both environmental and genetic risk factors. A variety of environmental risk factors, environmental carcinogens, and genetic predispositions greatly affect the risk of cancer [41]. Notably, the large heterogeneity of the human population in terms of dietary, lifestyle habits, and environmental exposures makes it difficult to assess the relationship between selected risk factors and cancer risk [24].

A number of studies have reported the effect of the environmental factor on disease risk to vary with genotype [53–56]. The *MMP* genetic polymorphisms may modify the significance of an environmental risk factor for bladder cancer through enhanced sensitivity to cigarette smoke [57–62]. Studies in cancer and other disease suggest that common genetic variants of *MMPs* may also interact with cigarette smoking in esophageal squamous cell carcinoma [63], lung cancer [64], and myocardial infarction [65]. However, despite this, there is still limited knowledge of both genetic and environmental causes of cancer. New studies are now required to explain the importance of common genetic variants in combinations with environmental factors and elucidate the existing dependence in cancerogenesis. In such cases, the challenge will be taking into account SNPs, CNVs, and their potential interactions with environmental risk factors for disease such as carcinogens and many others. It appears that the environmental exposure studies will use the next generation mutant mouse lines mimicking human genetic polymorphisms to examine their significance in human tumors [66].

## MMPs in carcinogenesis: genetic mouse models and human genetic association studies

As has been stated by Iyer et al., the creation of *Mmp*-deficient mice is one of the major MMP milestones [67]. The mouse model has been mainly selected from among the rodent model to study the role of MMPs.

The first cancer study using GEM models in *Mmp* was conducted in 1995 [68]. In the MMP studies, the most commonly used GEM models are knockout mice, double-deficient mice, but also transgenic mice. *Mmp*-deficient mice were also evaluated in induced disease such as arthritis, pulmonary fibrosis, and acute hepatitis [27] and in the mouse model of cardiovascular disease [69]. No physiological alterations were observed in the majority of *Mmp*-deficient mice, possibly due to the enzymatic compensation and other effects described by Scroyen et al. [70]. However, *Mmp*-deficient mice demonstrate the individual functions of MMPs [71].

Numerous studies in genetic mouse models of cancer suggest that MMP deficiency may lead to decreased or increased tumor progression, incidence, size, and metastasis. In most cases, experimental studies in genetic mouse models of cancer and human association studies confirmed the importance of MMPs (Table 1).

**Table 1:** Summary of MMP studies in genetic mouse models of cancer and genetic association studies which demonstrated significant association with cancer risk

Cancer type	Experimental genetic mouse models and human genetic association studies								
	MMPs with tumor-promoting effects				MMPs with tumor-suppressive effects	MMPs with promoting and antitumor effects			
	MMP-1	MMP-2	MMP-7	MT1-MMP	MMP-8	MMP-3	MMP-9	MMP-11	MMP-19
Bladder	[57, 58, 62]	[60]	[57]		[60]	[61]	[61]		
Breast, mammary gland		[72–75], [76]^a^	[78], [12, 77, 79, 80]^a^	[81]^a^		[82, 84], [68, 83]^a^	[79, 80]^a^	[85, 86]^a^	
Cervical		[87], [88]^a^					[88]^a^		
Colorectal, colon	[82, 89–91]	[92], [93]^a^	[93–95]^a^			[90, 96]	[93, 97]^a^		
Epithelial cell malignancy								[98]^a^	
Gastric		[99, 100]	[101–103]			[104]			
Head and neck, esophageal, oral cancer, tongue	[105–111]	[63, 112–114]	[103, 115]	[116]	[117]^a^				
Hepatocellular carcinoma				[118]					
Lung, melanoma	[64, 120], [119]^a^	[121, 122], [123, 124]^a^	[103], [123]^a^		[125]^a^		[123, 126]^a^		
Neuroblastoma							[127]^a^		
Osteosarcoma							[128]^a^		
Ovarian		[129]^a^	[130]				[129]^a^		
Pancreatic		[131]^a^	[132, 133]^a^	[134]^a^			[131, 135]^a^		
Prostate		[136]^a^	[136, 137]^a^				[136, 138]^a^		
Renal	[139, 140]								
Skin					[15]^a^				[141, 142]^a^
Squamous cell carcinoma							[143]^a^		
Various							[144]^a^		

^a^Studies using genetic mouse models

According to the results of the human clinical specimens, genetic mouse models, and human association studies, the role of MMPs in carcinogenesis may be categorized as follows: tumor-promoting, anticancer, and both effects [145].

### MMPs with tumor-promoting roles (MMP-1, MMP-2, MMP-7, MMP-14)

Genetic mouse model studies presenting the tumor-promoting role of MMPs in cancer are shown in Table 2. To our knowledge, only one study concerns *Mmp-1a*-deficient mouse ortholog of human *MMP-1*. In vivo data imply that *Mmp-1a* has a role in lung tumor progression [119]. Moreover, association studies suggest that functional polymorphism in the *MMP-1* is associated with risk of colorectal [82, 89–91], bladder [57, 58, 62], renal [139, 140], head and neck [105–111], and lung cancer [64, 120] and risk of lymph node metastases in breast cancer patients [146–148].

**Table 2:** The MMPs with tumor-promoting roles and functions in mouse models of cancer

Allelic composition mouse lines	References	Cancer type	Induction	Effect
MMP-1a, matrix metalloproteinase 1a, collagenase-1				
*Mmp-1a−/−* deficient mice	Fanjul-Fernández [119]	Lung	Exposure to urethane	Decreased tumor progression
MMP-2, matrix metalloproteinase 2, gelatinase A, collagenase IV				
*Mmp-2−/−* deficient mice	Itoh [124]	Lung, melanoma	Injection of B16-BL6, LLC cells	Decreased angiogenesis and tumor progression
*Rip1-Tag2;Mmp-2−/−* transgenic mice; deficient mice	Bergers [131]	Pancreatic		Decreased tumor size, did not contribute to development of angiogenic islets or tumor number
*HPV/E2;Mmp-2−/−* transgenic mice; deficient mice	Giraudo [88]	Cervical		Did not contribute to tumor incidence or volume, vascularity
*Mmp-2−/−* deficient mice	Acuff [123]	Lung	Injection of LLC cells	No differences in tumor incidence, tumor size
*Rag-1−/−;Mmp-2−/−* double-deficient mice	Kenny [129]	Ovarian	Injection of SKOV3ip1 cells	Did not alter cancer cell adhesion
*CR2-Tag;Mmp-2−/−* transgenic mice; deficient mice	Littlepage [136]	Prostate cancer lung and liver metastasis		Decreased tumor burden, prolonged survival, decreased lung metastasis, decreased blood vessel density
*Mmp-2−/−* deficient mice	Kitamura [93]	Colon cancer liver metastasis	Injection of CMT93 cells	Decreased tumor dissemination
*Mmp-2−/−* deficient mice	Thiolloy [76]	Breast to bone metastases	Injection of PyMT-Luc, 17L3C-Luc cells	Decreased bone resorption, contributes to mammary tumor-induced osteolysis, does not inhibit osteoclast precursor migration or osteoclastogenesis
MMP-7, matrix metalloproteinase 7, matrilysin-1, MAT				
*Min/+;Mmp7−/− Apc* ^Min^ mutation; deficient mice	Wilson [95]	Colon		Decrease of tumor multiplicity and tumor diameter
*MMTV/Mat;MMTV/neu* transgenic mice; transgenic mice	Rudolph-Owen [12]	Mammary gland		Expression contributes to early-stage mammary tumorigenesis
*Mmp-7−/−* deficient mice	Crawford [133]	Pancreatic ductal adenocarcinoma		Inhibited development of progressive metaplasia and acinar cell apoptosis
*Min/+;Mmp-7−/− Apc* ^Min^ mutation; deficient mice *Min/+/MMTV; Mmp-7−/−* transgenic mice; deficient mice	Hulboy [77]	Mammary gland	Injection of ENU	Influences early stage No effect on the development of tumor
*Rag-2−/−;Mmp-7*−/− double-deficient mice	Lynch [137]	Prostate	Tumor transplantation	Decreased tumor-induced osteolysis and RANKL processing
*Mmp-7−/−* deficient mice	Acuff [123]	Lung	Injection of LLC cells	Increased tumor incidence, no differences in tumor size
*MMTV-PyVT;Mmp-7−/−* transgenic mice; deficient mice	Martin [80]	Mammary gland lung metastasis		No differences in multifocal tumor incidence, no effect on the development of lung metastases
*Mmp-7−/−* deficient mice	Thiolloy [79]	Breast cancer bone metastasis	Injection of PyMT-Luc, 4T1-Luc cells	Contributes to tumor growth and tumor-induced osteolysis
*cis-Apc/Smad4;Mmp-7−/−* transgenic mice; deficient mice	Kitamura [94]	Colon	Injection of CMT93 cells	Decreased tumor incidence; MMPs are required for tumor formation but not for the invasion or fibrosis of SMAD4-dependent cancer
*Mmp-7−/−* deficient mice	Kitamura [93]	Colon cancer liver metastasis	Injection of CMT93 cells	No differences in tumor dissemination
*CR2-Tag;Mmp-7−/−* transgenic mice; deficient mice	Littlepage [136]	Prostate cancer lung and liver metastasis		Did not influence tumor growth, metastasis or survival
*Mmp-7−/−* deficient mice	Fukuda [132]	Pancreatic ductal adenocarcinoma	Injection of caerulein	Reduced tumor size and metastasis
MMP-14, membrane-type matrix metalloproteinase 1, MT1-MMP				
*MMTV/Mt1-mmp* transgenic mice	Ha [81]	Mammary gland abnormalities and adenocarcinoma		Overexpression of MT1-MMP-induced tumor formation
*Kras;Mt1-mmp Kras* mutation; transgenic mice	Krantz [134]	Pancreatic		Developed a greater number of large, dysplastic mucin-containing papillary lesions, pancreatic fibrosis

Konstantinopoulos et al. wrote in 2008 that MMP-2 may exert cancer-promoting effects [145], and also *Mmp-2*-deficient mice show antitumor effects on various cancers. The *MMP-2* genetic polymorphism was associated with risk of breast [72–75], gastric [99, 100], esophageal [63, 112], cervical [87], colorectal [92], lung [121, 122], head and neck [113, 114], and bladder cancer [60].

*Mmp-7* (matrilysin-1) has widely been studied in the deficient and transgenic mice; the results demonstrated influences on early-stage mammary cancer [12, 77] and decreased tumorigenesis [94, 95, 132, 137]. Studies on genetic polymorphism in the *MMP-7* show association with risk of bladder [57], breast [78], gastric [101–103], ovarian [130], head and neck [115], esophageal, and lung cancer [103].

MT1-MMP also known as MMP-14 has been described as MMP with anticancer effects [145], but two in vivo studies showed that overexpression of MT1-MMP-induced remodeling of the ECM and mammary gland adenocarcinoma formation [81]; in pancreatic cancer, the MT1-MMP overexpression was seen to affect cancer development [134]. The genetic polymorphism in the *MT1-MMP* showed association only with susceptibility to hepatocellular carcinoma [118] and oral cancer [116].

Moreover, the results of mouse studies concerning the influence of MMPs on carcinogenesis make it possible to draw some additional conclusions: *Mmp-1a* modulates immune response to chemical carcinogens by polarization of a Th1/Th2 [119], *Mmp-7* mediates tumor-induced osteolysis by solubilization of RANKL [79], and MT1-MMP may be able to increase TGF-β signaling [134].

### MMPs with tumor-suppressive roles (MMP-8)

The studies confirm that the MMP-8 (collagenase-2) is a metalloproteinase which may exert an anticancer effect (Table 3). Gene knockout mice have been also generated to distinguish the roles of *Mmp-8*. MMP-8 may control the invasion potential of tumor cells by modulating cell adhesion [125]. The protective role of MMP-8 has been shown in *Mmp-8*-deficient mice [15, 117]. *MMP-8* genetic polymorphism also showed association with cancer risk, i.e., with low risk of bladder cancer [60] and with lymph node metastases classification in breast cancer patients [149].

**Table 3:** The MMPs with tumor-suppressive roles and functions in mouse models of cancer

Allelic composition mouse lines	References	Cancer type	Induction	Effect
MMP-8, matrix metalloproteinase 8, collagenase-2				
*Mmp-8−/−* deficient mice	Balbín [15]	Skin	Exposure to DMBA, TPA	Increased the incidence of tumors
*Mmp-8−/−* deficient mice	Gutiérrez-Fernández [125]	Lung	Injection of B16F10, LLC cells	Increased the metastasis formation
*Mmp-8−/−* deficient mice	Korpi [117]	Squamous cell carcinoma of the tongue	Exposure to 4NQO	Increased the incidence of tumors

### MMPs with promoting and antitumor-promoting roles (MMP-3, MMP-9, MMP-11, MMP-19)

Genetic mouse models of cancer presents a dual role of MMPs in carcinogenesis, especially for the MMP-3 and MMP-9 (Table 4). MMP-3 is also known as stromelysin-1 and STR1. *WAP-Str1* transgenic mice in mammary cancer can influence the initiation of a tumor; on the other hand, there were no differences in mammary tumor invasion in *MMTV/TGF-α;Str1* transgenic mice [68, 83]. Genetic polymorphism in the *MMP-3* is associated with bladder [61], breast [82, 84], head and neck [104], and colorectal cancer [90, 96].

**Table 4:** The MMPs with promoting and antitumor-promoting roles and functions in mouse models of cancer

Allelic composition mouse lines	References	Cancer type	Induction	Effect
MMP-3, matrix metalloproteinase 3, stromelysin-1, STR1				
*MMTV/TGF-α;Str1* transgenic mice; transgenic mice	Witty [68]	Mammary	Exposure to DMBA	Expression is not sufficient to confer invasive and metastatic potential
*WAP-Str1* transgenic mice	Sternlicht [83]	Mammary		Influences tumor initiation and alters neoplastic risk
MMP-9, matrix metalloproteinase 9, gelatinase B				
*Mmp-9−/−* deficient mice	Itoh [126]	Lung, melanoma	Injection of B16-BL6, LLC cells	Important role in the process of tumor metastasis
*HPV16;Mmp-9−/−* transgenic mice; deficient mice	Coussens [143]	Squamous cell carcinoma		Decreased tumor incidence, reduced epithelial hyperproliferation at all stages of carcinogenesis and tumors arising are more malignant
*Rip1-Tag2;Mmp-9−/−* transgenic mice; deficient mice	Bergers [131]	Pancreatic		Decreased tumor number and size, developed fewer angiogenic islets
*HPV/E2;Mmp-9−/−* transgenic mice; deficient mice	Giraudo [88]	Cervical		Decreased tumor incidence Reduction of tumor volume Reduced vascularity
*Rag-1−/−;Mmp-9−/−* double-deficient mice	Jodele [127]	Neuroblastoma	Tumor xenotransplantation	Contributes to the recruitment of bone marrow-derived cells to the tumor microenvironment
*Rag-2−/−;Mmp-9−/−* double-deficient mice *Mmp-9−/−* deficient mice	Acuff [123]	Lung	Injection of LUC-A549 cells Injection of LLC cells	Decreased tumor incidence, more apoptosis, contributes to the early establishment of tumors and not to tumor growth No differences in tumor incidence, tumor size
*MMTV-PyVT;Mmp-9−/−* transgenic mice	Martin [80]	Mammary gland lung metastasis		Decrease in lung tumor incidence, angiogenesis, no differences in multifocal tumor incidence
*Rag-1−/−; Mmp-9−/−* double-deficient mice	Kenny [129]	Ovarian	Injection of SKOV3ip1 cells	Did not alter cancer cell adhesion
*Mmp-9−/−* deficient mice	Ahn [144]	Various	Injection of MT1A2, TG1-1, RIF, B16F1, and LLC cells	Decreased tumor growth, abrogated tumor vasculogenesis
*Mmp-9−/−* deficient mice	Kubota [128]	Osteosarcoma	Tumor transplantation	Decreased tumor growth
*Mmp-9−/−* deficient mice	Thiolloy [79]	Breast cancer, bone metastasis	Injection of PyMT-Luc, 4T1-Luc cells	Did not contribute to tumor growth or tumor-induced osteolysis
*CR2-Tag; Mmp-9−/−* transgenic mice; deficient mice	Littlepage [136]	Prostate cancer, lung and liver metastasis		Did no influence tumor growth, metastasis or survival
*Mmp-9 −/−* deficient mice	Kitamura [93]	Colon cancer liver metastasis	Injection of CMT93 cells	Decreased tumor dissemination
*Mmp-9−/−* deficient mice	Garg [97]	Colitis-associated colon cancer	Injection of AOM, DSS	Increased susceptibility to colitis-associated colon cancer
*Rag-2−/−;Mmp-9−/−* double-deficient mice	Bruni-Cardoso [138]	Prostate tumor progression in the bone	Tumor transplantation	Decreased angiogenesis
*Myc-BclXl; Mmp-9−/−* transgenic mice; deficient mice *Rip1-Tag2; Mmp-9−/−* transgenic mice; deficient mice	Shchors [135]	Pancreatic neuroendocrine		Increased tumor invasion
MMP-11, matrix metalloproteinase 11, stromelysin-3, ST3				
*St3−/−* deficient mice	Masson [98]	Epithelial cell malignancy	Exposure to DMBA	Decreased tumorigenesis and sensitivity to carcinogens
*MMTV-ras;St3−/−* transgenic mice; deficient mice	Andarawewa [86]	Mammary gland		Increased carcinoma, developed more metastases
*St3−/−* deficient mice	Andarawewa [85]	Breast	Injection of C26 cells	Negatively regulates fat homeostasis
MMP19, matrix metalloproteinase 19				
*Mmp-19−/−* deficient mice	Pendás [141]	Skin	Injection of MCA	Decreased susceptibility, resistant to the development induced fibrosarcomas
*Mmp-19−/−* deficient mice	Jost [142]	Skin	PDVA cell implantation	Increased tumor invasion

MMP-9 is probably the most widely studied metalloproteinase. Studies show that *Mmp*-9 is very important in tumor incidence and metastasis [80, 88, 93, 123, 126–128, 131, 138, 143, 144] and may have an anticancer effect in colitis-associated colon cancer and pancreatic neuroendocrine tumor [97, 135]. Association was found between the *MMP-9* polymorphisms and risk of bladder cancer [61], and tumor stage or grade [59]. Additionally, studies of genetic mouse models describe the roles of MMP-9 release VEGFA from the extracellular matrix [131], change Notch-1 activation by module cell cycle inhibitor p21/WAF1/Cip1 and beta-catenin protein activity [97], and participate in keratinocyte differentiation [143]. Expression of MMP-9 was suppressed by zoledronic acid [88].

MMP-11 (stromelisin-3 or ST3) and MMP-19 are important in cancer cell proliferation [150] and demonstrated the opposite roles in studies of genetic mouse models. MMP-11 has influence on adipogenic markers such as peroxisome proliferator-activated receptor and adipocyte protein 2 [85]. The *Mmp-19*-deficient mouse model not only is considered to negatively regulate the early steps of tumor angiogenesis and invasion but is also thought to be associated with decreased susceptibility to cancer [141, 142]. The association of genetic polymorphisms in the *MMP-11* and *MMP-19* with cancer risk has not been investigated; only the results of MMP-19 expression have been described as associated with cancer processes [151–153].

Tumorigenic phenotypes in mice were induced either by chemical induction, transgenic complementation, tumor cell injection, or tumor transplantation. The tumors were chemically induced in mice by exposure to selected carcinogens, like 7,12-dimethylbenzanthracene (DMBA) [68, 98], urethane (ethyl carbamate) [119], 4-nitroquinoline-*N*-oxide (4NQO) [117], methylcholanthrene (MCA) [141], *A*′-ethyl-*A*′-nitrosourea (ENU) [77], DMBA also with tumor promoter 12-*O*-tetradecanoylphorbol-13-acetate (TPA) [15], and potent carcinogen azoxymethane (AOM) and dextran sodium sulfate (DSS) [97].

The researchers in in vivo studies of MMPs also used transgenic complementation of *Mmp*-deficient mice or mutation necessary for cancer. The various transgenic and mutation mouse models of cancer were used: models of pancreatic carcinoma—*Rip1*-*Tag2* [131], *Kras* [134], *Myc-BclXl* [135]; model of cervical cancer—*HPV/E2* [88]; model of prostate cancer—*CR2-Tag* [136]; model of colon cancer—*cis-Apc/Smad4* [94]; model of squamous cell carcinoma—*HPV16* [143]; model of intestinal neoplasia—*Min/Apc* [95]; and models of mammary cancer—*MMTV* [12, 68, 81], *MMTV-PyVT* [80], and *MMTV-ras* [86]. Also, five studies used immunodeficient mice *Rag-1* [127, 129] and *Rag-2* [123, 137, 138].

The primary tumors were generated by the injection of tumor cells such as osteolytic luciferase-tagged mammary tumor cell lines (PyMT-Luc and 4T1-Luc, 17L3C-Luc) [76, 79], CMT93 mouse colon cancer cells [93, 94], SKOV3ip1 cells [129], LUC-A549 cells [123], Lewis lung carcinoma cells (LLC) [123–126, 144], and B16F10 cells [125], B16-BL6 cells [126], and others [85, 142, 144]. Lynch et al., Jodele et al., Kubota et al., and Bruni-Cardoso et al. have used tumor transplantation or xenotransplantation into *Mmp-*deficient mice [127, 128, 137, 138].

Different strain-specific responses occurring in mice with various genetic backgrounds may exert different effects in carcinogenesis. Therefore, only specific selected strains should be used in the experiments [32].

## Conclusion

Already in 1999, Westermarck and Kahari (in review) described in vitro and in vivo studies and reported the evidence for the role and biological mechanisms of the MMPs driving tumor invasion and growth [35]. Currently, the results of the genetic mouse studies demonstrated that deficient mice and transgenic mice models are a successful tool used to identify and explain the functions of MMPs. These studies confirm the importance of differences in genetic pathophysiological mechanisms for distinct *MMP* genes in various cancer subtypes. In genetic mouse models in which the tumors are induced by chemical carcinogens, cell injection, or tumor implantation, the changing of genetic background may affect tumor susceptibility.

Accumulating evidence suggests that susceptibility to cancer is mediated by genetic and environmental factors and complex gene-environment interactions. Therefore, there is an urgent need for mouse studies in which we may simplify experiments by control of variables such as dietary and lifestyle habits and environmental exposures. It seems that the loss of function of the *Mmp* gene may be comparable to the effect that occurs for the functional genetic polymorphism in the *MMP*. Therefore, the GEM can be used as a background for showing a relationship between environmental risk factors, genotype, and cancer.

In this review, we summarize and compare the results of genetic mouse models and human association studies, already categorized according to the possible effect of MMPs on the development of cancer. Among the known 25 MMPs, only 9 MMPs have been examined in mouse models: MMP-1, MMP-2, MMP-3, MMP-7, MMP-8, MMP-9, MMP-11, MMP-14, MMP-19. Presented studies confirm that one of the most widely studied of the MMPs is MMP-9. Moreover, only the breast, mammary gland, lung, and pancreatic cancer sites have been extensively studied in mice. Therefore, it seems to be important to focus on the role of various MMPs in other types of cancer, including bladder cancer.

It should be noted that to gain a better understanding of the role of various MMPs in various cancer types, each of the MMPs should be assessed within the same tumor environment. Therefore, more in vivo and in vitro studies are required to allow comparison of the same cancer types and stages. Results from these studies which are based on fundamental knowledge are essential for further investigation in human cancer association studies.

Taken together, genetic in vivo studies complemented by human genetic association studies will extend our knowledge about the genetic predisposition to cancer, by clarifying some of the problems mentioned above: the genetic modifiers and gene-environment interaction. Future studies will enable the identification of genetic markers essential for early detection of tumors in the future by means of molecular diagnostic procedures.
